# Supplementary material for: Mechanisms governing the pioneering and redistribution capabilities of the non-classical pioneer PU.1
Source: Nat Commun. 2020 Jan 21;11:402. doi: 10.1038/s41467-019-13960-2 (PMC6972792; doi:10.1038/s41467-019-13960-2)
Supplement: Supplementary file 7 — Source data [file 41467_2019_13960_MOESM7_ESM.zip › Source_Data/FigureS5/FigureS5A_MotifsCommonPeaks/homerResults/motif29.similar.html]

motif29

## Information for motif29

G
C
T
A
C
T
G
A
A
G
T
C
T
C
G
A
C
A
T
G
G
T
A
C
G
C
A
T
C
T
A
G
  
Reverse Opposite:  

G
A
T
C
C
G
T
A
C
A
T
G
G
T
A
C
A
C
G
T
T
C
A
G
G
A
C
T
C
G
A
T
  

|  |  |
| --- | --- |
| p-value: | 1e-51 |
| log p-value: | -1.177e+02 |
| Information Content per bp: | 1.609 |
| Number of Target Sequences with motif | 2412.0 |
| Percentage of Target Sequences with motif | 27.14% |
| Number of Background Sequences with motif | 7488.5 |
| Percentage of Background Sequences with motif | 19.70% |
| Average Position of motif in Targets | 101.2 +/- 56.3bp |
| Average Position of motif in Background | 100.7 +/- 65.8bp |
| Strand Bias (log2 ratio + to - strand density) | -0.0 |
| Multiplicity (# of sites on avg that occur together) | 1.13 |
| Motif File: | file (matrix) reverse opposite |

### Similar de novo motifs found

|  |  |  |  |  |  |  |  |
| --- | --- | --- | --- | --- | --- | --- | --- |
| Rank | Match Score | Redundant Motif | P-value | log P-value | % of Targets | % of Background | Motif file |
| 1 | 0.789 | G C A T C T A G T A G C A G T C C G T A A C T G A G C T G A C T C T A G | 1e-45 | -104.261593 | 19.13% | 13.09% | motif file (matrix) |
| 2 | 0.873 | C G T A C T A G G T A C C G T A C T A G A G T C G A C T A C T G A G T C | 1e-35 | -81.214614 | 15.91% | 10.99% | motif file (matrix) |
| 3 | 0.845 | T A C G T A G C G C T A C T G A T A G C C G T A C A T G T G A C C G A T T A C G G A C T | 1e-26 | -61.587062 | 5.17% | 2.78% | motif file (matrix) |
| 4 | 0.761 | A G T C C G T A A T C G G T C A A G C T A T C G A C G T C A G T A C T G | 1e-18 | -42.086150 | 4.12% | 2.34% | motif file (matrix) |
